# Rhabdomyolysis and acute renal failure after strenuous exercise and alcohol abuse: case report and literature review

**DOI:** 10.1590/S1516-31802005000100008

**Published:** 2005-01-02

**Authors:** Elizabeth De Francesco Daher, Geraldo Bezerra da Silva, Denise Menezes Brunetta, Lícia Borges Pontes, Glaydcianne Pinheiro Bezerra

**Keywords:** Rhabdomyolysis, Acute kidney failure, Compartment syndrome, Exercise, Alcoholic intoxication, Rabdomiólise, Insuficiência renal aguda, Síndromes de compartimento, Exercícios, Intoxicação por álcool

## Abstract

**CONTEXT:**

Rhabdomyolysis is a severe and lifethreatening condition in which skeletal muscle is damaged. Acute renal failure due to rhabdomyolysis has been widely described and its main pathophysiological mechanisms are renal vasoconstriction, intraluminal cast formation and direct myoglobin toxicity.

**OBJECTIVE:**

To report on a case of acute renal failure (ARF) induced by rhabdomyolysis due to strenuous exercise and alcohol abuse and to describe the pathophysiology of this type of ARF.

**CASE REPORT:**

A 39-year-old man arrived at the hospital emergency service with swollen legs and lower extremity compartment syndrome. He was oliguric and had serum creatinine and urea levels of 8.1 mg/dl and 195 mg/dl, respectively. The diagnosis of rhabdomyolysis was made through clinical and laboratory findings (creatine kinase activity of 26320 IU/l). The initial treatment consisted of fluid replacement and forced diuresis. The specific treatment for compartment syndrome, such as fasciotomy, was avoided in order to prevent infection. Partial recovery of renal function was recorded, after ten hemodialysis sessions. Complete recovery was observed after two months of follow-up.

## RESUMO

**CONTEXTO:**

Rabdomiólise é uma condição clínica severa e ameaçadora à vida, em que ocorre destruição de músculo esquelético. Insuficiência renal aguda por rabdomiólise já foi bem descrita, sendo os principais mecanismos fisiopatológicos vasoconstrição renal, formação de depósitos intratubulares e toxicidade direta da mioglobina.

**TIPO DE ESTUDO:**

Relato de caso e revisão da literatura.

**OBJETIVO:**

Relatar um caso de insuficiência renal aguda (IRA) induzida por rabdomiólise devida a exercícios físicos intensos e uso abusivo de álcool e descrever os principais mecanismos fisiopatológicos deste tipo de IRA.

**RELATO DE CASO:**

Homem de 39 anos foi admitido na emergência com edema e síndrome compartimental em membros inferiores. Ele estava oligúrico, e os níveis séricos de creatinina e uréia eram de 8,1mg/dl e 195mg/dl, respectivamente. O diagnóstico de rabdomiólise foi obtido através dos dados da história clínica e dos exames laboratoriais (creatinoquinase de 26320 UI/l). O tratamento inicial consistiu em reposição volêmica e diurese forçada. Tratamento específico para síndrome compartimental, como fasciotomia, não foi realizado para que se evitasse a ocorrência de infecções. Recuperação parcial da função renal foi observada após 10 sessões de hemodiálise. Recuperação completa ocorreu após dois meses de acompanhamento.

## INTRODUCTION

Rhabdomyolysis is a life-threatening condition in which skeletal muscle is damaged. There are numerous causes, and these include the use of drugs, muscle trauma, exposure to toxins, infections, hyperthermia, seizures and electrolyte abnormalities leading to cell lysis through ischemia and acidosis.^1-4^ Acute renal failure (ARF) due to rhabdomyolysis has been widely described in different clinical settings, and its main pathophysiological mechanisms are renal vasoconstriction, intraluminal cast formation and direct myoglobin toxicity. These have already been demonstrated *in vitro*.^1,5^ Rhabdomyolysis is an important cause of ARF and remains unrecognized in many cases.^1,6,7^ Around 33% of the episodes of rhabdomyolysis lead to ARF.^8^ Strenuous exercise may lead to the disintegration of striated muscle, thereby resulting in the release of muscle cell constituents into the extracellular fluid and circulation. This may consequently cause pigment-nephropathy and ARF.^9,10^

Many patients presenting with muscle swelling and compartment syndrome have their diagnosis confounded with deep vein thrombosis, thus delaying the correct treatment.^11^ The occurrence of muscle swelling and a history of alcohol misuse, drug overdose, traumatic muscle lesions and prolonged immobility or unconsciousness should raise the suspicion of rhabdomyolysis.^11-13^

The diagnosis is based on clinical features and laboratory findings such as urine stick reagents testing positive for blood in the absence of urinary erythrocytes; myoglobinuria and serum creatine kinase levels raised to more than five times the normal upper limit; high levels of lactate dehydrogenase, aspartate and alanine aminotransferases, phosphate and potassium; and initially low serum calcium concentration.^7,11^

The treatment should be instituted immediately, in order to prevent the factors that cause ARF, such as volume depletion, tubule obstruction, aciduria and free radical release.^1^ There is no specific treatment stated for rhabdomyolysis, but it is advised to give vigorous fluid replacement and sometimes administer mannitol to maintain adequate urine flow. Alkalization of urine by using sodium bicarbonate can reduce the risk of tubule obstruction by myoglobin casts.^7^ The prognosis may be excellent if the underlying mechanism of rhabdomyolysis can be identified and reversed, whenever this is possible.^7^

After obtaining the patient’s consent, we report on the case of a man who developed rhabdomyolysis after exhausting exercise followed by a heavy bout of drinking alcohol. He arrived at the emergency department with swollen legs and lower extremity compartment syndrome.

## CASE REPORT

A 39-year-old man had been practicing running four kilometers, using halfkilogram weights attached to each leg, on three days per week for 20 days. The day after the last race, he started to drink large quantities of red wine, continuing for two days. After this bout of drinking, he had a fight with his brother, who weighs 80 kg. His brother fell on top of him, and he then lost consciousness for 10 hours, lying on a hard sofa. When he woke up, his legs were extremely painful, he could not walk, and he had a mild headache. According to his brother, he had a kind of seizure. The next day, he went to a small healthcare center, from which he received symptomatic medications, and then went back home.

**Figure 1:**
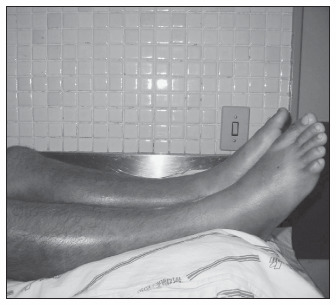
Patient’s swollen legs, with tense calves at admission.

A few hours later, he was admitted to the emergency room of the General Hospital of Fortaleza with painful, swollen, tense and tender calves (Figure 1), and with signs of lower extremity compartment syndrome and oliguria-anuria, presenting with dark brown urine. No pulse could be felt in his feet. The physical examination also showed the presence of ecchymotic lesions on his left arm and buttock. Serum creatinine of 8.1 mg/dl and urea of 195 mg/dl were recorded. Urinalysis using a dipstick was positive for red blood cells, white blood cells and proteins. Vigorous fluid replacement was undertaken, and forced diuresis was attempted through the administration of furosemide. His blood pressure was 160/100 mmHg, and antihypertensive therapy using nifedipine and captopril was administered. Rhabdomyolysis was suspected and a creatine kinase activity level of 26320 IU/l was found. The laboratory findings during his hospital stay are shown in Table 1. Acute renal failure was evident and hemodialysis was started. He was found to have a history of previous high blood pressure and alcohol and cocaine abuse.

**Table 1:** Laboratory findings during hospitalization of a 39 year-old male patient with suspected rhabdomyolysis

Day	1	2	3	4	5	9	12	15	18	23	27	30	33	40	45
Urea (mg/dl)	195	243	184	123	124	118	95	91	109	33	40	31	44	–	35
Creatinine (mg/dl)	8.1	9.9	10.1	7.7	7.3	5.1	3.5	2.8	3.1	0.8	0.5	1.1	0.6	–	1.0
Potassium (mEq/l)	4.9	7.6	5.5	4.4	4.3	4.0	4.7	4.6	4.6	4.8	4.7	4.2	6.4	–	4.4
Sodium (mEq/l)	129	126	133	133	129	128	135	133	133	133	131	132	137	–	138
Ionic Calcium*	1.03	0.90	1.06	1.14	–	1.38	–	1.36	–	1.25	1.28	1.34	1.18	–	1.32
Uric acid (mg/dl)	–	12.5	–	7.7	–	–	6.5	5.5	–	–	–	–	–	–	–
Hematocrit (%)	32.5	30.5	26.9	24.8	20.2	19.9	18.9	22.8	20.9	27.1	26.6	26.6	27.6	34.0	30.3
Hemoglobin (g/dl)	11.4	10.0	8.93	8.29	6.80	6.68	6.31	7.51	6.98	8.93	9.1	9.1	8.53	11.0	10.1
White blood cells (x 10^3^/mm^3^)	9.3	8.82	7.98	7.6	7.47	7.95	14.4	8.15	9.03	10.9	10.9	10.9	5.09	7.04	13.2
Platelets (x 10^3^/mm^3^)	148	136	133	145	152	271	132	112	128	339	438	438	382	371	–
Prothrombin time**	11.0	–	12.5	12.4	–	–	–	–	–	–	14.6	13.9	–	–	–
Partial thromboplastin time***	28.1	–	36.9	52.2	–	–	–	–	–	–	22.6	31.7	–	–	–
AST (IU/l)	–	730	–	–	293	75	38	67	67	74	–	–	–	–	–
ALT (IU/l)	–	480	–	–	256	134	–	–	–	–	–	–	–	–	–
Direct bilirubin (mg/dl)	–	–	0.07	–	0.20	–	–	–	–	–	–	–	–	–	–
Indirect bilirubin (mg/dl)	–	–	0.59	–	0.98	–	–	–	–	–	–	–	–	–	–
LDH (IU/l)	–	3280	3447	–	–	–	1065	–	–	–	–	–	–	–	–
CK (IU/l)	–	26320	39420	20570	–	1740	653	202	202	105	–	149	125	–	–
Urine – Red blood cells	–	+++	+++	–	–	+++	+	+	–	–	–	–	–	–	–
White blood cells	–	+++	+++	–	–	+++	+++	++	–	–	–	–	–	–	–
Protein	–	+++	+++	–	–	+++	++	+	–	–	–	–	–	–	–

*AST: aspartate amino transaminase; ALT: alanine amino transaminase; LDH: lactate dehydrogenase; CK: creatine kinase;*

* *normal ionic calcium levels: 1.12-1.32 mEq/l*

** *control for prothrombin time = 13"*

*** *control for partial thromboplastin time = 28".*

**Figure 2:**
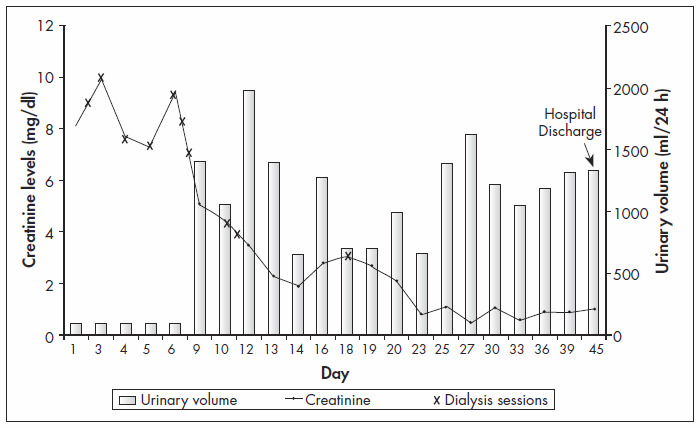
Urinary volume, serum creatinine levels and dialysis sessions during hospitalization, for a 39 year-old male patient with suspicion of rhabdomyolysis.

On the seventh day of hospitalization, he presented with great pain and paresthesia in his feet. Physiotherapy was instituted in order to recover the movement in his legs. After ten sessions of hemodialysis, the urine volume started to increase, and the levels of urea and creatinine decreased to normal levels (Figure 2).

He was discharged from hospital after 45 days, still with painful legs and with difficulty in walking. At a follow-up evaluation, two months later, he presented with great improvement of the movement in his legs, and complete recovery of renal function was recorded.

## DISCUSSION

Rhabdomyolysis is characterized by a broad spectrum of manifestations, ranging from a subclinical rise in creatine kinase to a medical emergency comprising interstitial and muscle cell edema, reduction in intravascular volume and pigment-induced acute renal failure.^1^ Myoglobinuria symptoms include muscle pain and weakness, pigmented urine and brown casts in tubules.^5^

After the occurrence of muscle damage, myoglobin is released into the circulation and is easily filtered by the glomeruli, with a depuration rate that corresponds to 75% of creatinine clearance.^14^ The serum levels of myoglobin can remain normal while urinary levels are high, because of this rapid filtration. There are two factors that predispose towards acute renal failure (ARF) due to rhabdomyolysis: hypovolemia secondary to liquid loss from damaged muscles and presence of acidic urine.^14^ In the absence of these events, myoglobin has a less nephrotoxic effect.^6,14^

The pathophysiology of myoglobinuric ARF has been extensively studied in an animal model of glycerol-induced ARF.^1,6,15^ The main mechanisms involved are renal vasoconstriction, intraluminal cast formation and direct heme protein-induced cytotoxicity.^6,14^ Renal vasoconstriction is favored by muscle necrosis, which leads to hypovolemia and the activation of cytokines. This increases capillary permeability and the binding of heme protein to nitric oxide: the endothelium relaxing factor.^14^ Casts are produced after the filtration of myoglobin through the glomerular basement membrane, which causes water reabsorption and a rise in myoglobin concentration. Following this, in the presence of acidic urine, myoglobin precipitation takes place and causes obstructive cast formation.^1^ Dehydration and renal vasoconstriction favor this process, through increased tubule reabsorption of sodium and water, which consequently increases myoglobin concentration in the tubules.^14^

Some studies have described the occurrence of dilation in the renal tubules, thus suggesting a high pressure in these compartments and contributing towards decreasing glomerular filtration.^16,17^ However, in the glycerol model of ARF due to rhabdomyolysis, it has been demonstrated that many tubules collapse and the mean intratubule pressure is below normal values.^15^ The high rates of generation and urinary excretion of uric acid further contribute towards tubule obstruction by uric acid casts.^1^

One additional factor that favors the precipitation of myoglobin and uric acid is low pH in the tubule urine, which is common because of underlying acidosis.^1^ It has been demonstrated that when urinary pH was kept at 8.0, 78% of the myoglobin was excreted by the kidneys.^6^ Myoglobin can, through the heme fraction, induce the release of free iron, which catalyses free radical production and further enhances ischemic tubule damage.^6,14^ Alkaline conditions prevent this effect by stabilizing the reactive ferryl myoglobin complex.^1^ The heme fraction can also initiate lipid peroxidation and renal tubule injury.^18^ In an experimental study, the earliest renal changes caused by myoglobin were diminished by pyruvate-stimulated gluconeogenesis, decreased total glutathione levels and induced lipid peroxidation. Pretreatment using reduced glutathione has provided complete protection of kidney slices from myoglobin toxicity.^5^

Another factor that could increase tubule obstruction is the possible occurrence of disseminated intravascular coagulopathy that has been described in rhabdomyolysis.^7,19,20^ This event causes the release of thromboplastin, leading to the production of microthrombus in the glomeruli and consequently decreased glomerular filtration.^14^

In the case presented here, we could see an elevated creatine kinase level and biochemical abnormalities such as raised levels of aspartate amino transaminase (AST) and alanine amino transaminase (ALT), lactate dehydrogenase, potassium and uric acid, accompanied by a low level of serum ionic calcium, as demonstrated in Table 1. These findings strongly suggest rhabdomyolysis.^1,11^ Our patient had his muscles damaged by different agents, including alcohol abuse and strenuous exercise. The fact that he was doing exercises using weights attached to his legs was crucial to the muscle injury. The main muscles forced were those in the legs, which led to the development of lower extremity compartment syndrome.

Compartment syndrome is defined as a condition in which increased pressure within a limited space impairs capillary perfusion of the tissue within that space and can cause rhabdomyolysis.^21^ In the case presented here, the increase in compartment pressure seems to have been caused by the muscle injury, which led to edema formation and impairment of the normal circulation. The high pressure in his legs could be seen at admission through visible edema in his calves, which were stiff upon palpation (so-called doughy muscle), and severe pain.

Strenuous muscular exercise seems to play a decisive role in the pathogenesis of rhabdomyolysis, especially in untrained subjects or in individuals experiencing extremely hot or humid conditions.^7,9,22^ Among military recruits, intensive training in hot weather may predispose to exertion heat stroke and rhabdomyolysis.^10^ High temperatures are very relevant in the present case, because our city (Fortaleza) has very hot weather, with a mean annual temperature of 33ºC. Our patient had been running under such temperatures for several hours.

According to Vanholder et al.,^1^ patients with acute or chronic alcohol intoxication have muscle dysfunction due to a combination of immobilization, hypokalemia, hypophosphatemia, agitation and direct myotoxicity of ethanol. Electrolyte abnormalities caused by alcohol ingestion are also important in muscle damage. Ethanol intoxication entails water-electrolyte and acid-base imbalance, in the form of metabolic acidosis, hypomagnesemia, hypocalcemia and hypophosphatemia. These are thought to take part in the mechanism for cell lysis.^4^ Alcohol has been associated with rhabdomyolysis in patients from Japan. All of these patients had been unconscious for several hours, and this contributed towards rhabdomyolysis through pressure necrosis, leading to acute renal failure in one of them.^12,13^ This could be observed in our patient, who had been drinking large amounts of alcohol for more than twenty years and had also undergone a heavy bout of drinking one day before the symptoms started, which led to unconsciousness for approximately 10 hours.

The patient said he had stopped using cocaine some months before this clinical event, but we must raise the suspicion of cocaine-induced rhabdomyolysis. In one study performed by Welch et al.,^23^ 24% of cocaine users treated in the emergency room had rhabdomyolysis. Cocaine use was responsible for acute renal failure without rhabdomyolysis in one Spanish patient.^24^ The creatine kinase level of this patient was 107 UI/l and the only suggested cause of renal failure was the use of cocaine, through an underlying mechanism of intense vasoconstriction. Our patient used cocaine for eight years and could even have been still using it. However, it was not possible to assay the serum cocaine level to confirm our hypothesis of recent cocaine use. A wide spectrum of renal complications can occur with both acute and chronic use of this substance. Rhabdomyolysis is very common after cocaine use, because of prolonged vasoconstriction that causes muscle ischemia.^25^ Hypertension was found in our patient’s history. Blood pressure frequently rises after using cocaine and this appears to contribute towards the development of ischemic renal failure.^26,27^

The diagnosis of rhabdomyolysis is achieved mainly through clinical features, including any history of strained exercise and alcohol use. The urine has a typical reddish-brown color, even in the absence of hematuria, and the main laboratory finding is an increased serum creatine kinase level. Rhabdomyolysis is easily confounded with deep vein thrombosis when the patient presents with acutely swollen and painful legs and an absence of pulse in the foot.^11^ All these were seen in our patient, but the history of running in an unusually forced manner and the episode of alcohol abuse lasting for two consecutive days made us think of rhabdomyolysis as the first diagnostic hypothesis. This was essential for the initial correct management of the patient, with the avoidance of the use of contrast media for diagnosing vessel obstruction, which would have caused even more renal damage.

No randomized trials for the management of rhabdomyolysis have yet been conducted, but there is a consensus for the administration of intravascular volume expansion by using saline solution and mannitol, to maintain urine output at more than 200-300 ml/hour.^7^ Alkalization of urine can be done in order to reduce the formation of myoglobin casts in renal tubules.^7^ Homsi et al.,^28^ in a retrospective study, showed that progression to established renal failure following rhabdomyolysis could be totally avoided with prophylactic treatment in which volume repletion was achieved using saline alone, and the use of bicarbonate and mannitol was unnecessary. The treatment administered to our patient consisted initially of vigorous fluid replacement and forced diuresis using furosemide. The urea and creatinine levels were increasing, and therefore dialysis treatment was necessary. After ten hemodialysis sessions, his renal function had been restored, and this could be confirmed by the decrease in serum creatinine and the normal diuresis.

Specific treatment for compartment syndrome by means of fasciotomy is controversial. Some physicians advise surgical intervention, through immediate decompression of the muscles, thereby decreasing the pressure. However this creates a potential source of infection.^1,29^ In our case, fasciotomy was avoided and conservative therapy was instituted. Such a choice was deemed successful by Robinson et al.,^30^ who studied six athletes with high compartment pressure. They were simply monitored in the hospital for muscle necrosis and acute renal failure. All these patients recovered completely.

In summary, the rhabdomyolysis described here was caused by different factors that had a cumulative effect and all contributed towards the development of acute renal failure. The diagnosis was achieved through clinical evidence and laboratory findings and the administration of contrast media was avoided in order to protect his kidneys. Fasciotomy was not performed, because it could have created a potential source of infection. Hemodialysis was the key component of the treatment and complete recovery of renal function was recorded two months after hospital discharge.
